# Drug repositioning pipeline integrating community analysis in drug-drug similarity networks and automated ATC community labeling to foster molecular docking analysis

**DOI:** 10.3389/fbinf.2025.1666716

**Published:** 2025-10-23

**Authors:** Daiana Colibăşanu, Vlad Groza, Maria Antonietta Occhiuzzi, Fedora Grande, Mihai Udrescu, Lucreția Udrescu

**Affiliations:** 1 Center for Drug Data Analysis, Cheminformatics, and the Internet of Medical Things, Victor Babeş University of Medicine and Pharmacy Timişoara, Timişoara, Romania; 2 Department II-Pharmaceutical Chemistry, Victor Babeş University of Medicine and Pharmacy Timişoara, Timişoara, Romania; 3 Department of Computer and Information Technology, Politehnica University Timişoara, Timişoara, Romania; 4 Department of Pharmacy, Health and Nutritional Sciences, University of Calabria, Rende, Italy; 5 Department I-Drug Analysis, Victor Babeş University of Medicine and Pharmacy Timişoara, Timişoara, Romania

**Keywords:** drug repositioning, drug-disease network, drug-drug similarity network, ATC labeling, molecular docking

## Abstract

**Introduction:**

Drug repositioning—finding new therapeutic uses for existing drugs—can dramatically reduce development time and cost, but requires efficient computational frameworks to generate and validate repositioning hypotheses. Network-based methods can uncover drug communities with shared pharmacological properties, while molecular docking offers mechanistic insights by predicting drug–target binding.

**Methods:**

We introduce an end-to-end, fully automated pipeline that (1) constructs a tripartite drug-gene-disease network from DrugBank and DisGeNET, (2) projects it into a drug-drug similarity network for community detection, (3) labels communities *via* Anatomical Therapeutic Chemical (ATC) codes to generate repositioning hints and identify relevant targets, (4) validates hints through automated literature searches, and (5) prioritizes candidates *via* targeted molecular docking.

**Results:**

After filtering for connectivity and size, 12 robust communities emerged from the initial 34 clusters. The pipeline correctly matched 53.4% of drugs to their ATC level 1 community label *via* database entries; literature validation confirmed an additional 20.2%, yielding 73.6% overall accuracy. The remaining 26.4% of drugs were flagged as repositioning candidates. To illustrate the advantages of our pipeline, molecular docking studies of chloramphenicol demonstrated stable binding and interaction profiles similar to those of known inhibitors, reinforcing its potential as an anticancer agent.

**Conclusion:**

Our integrated pipeline effectively integrates network-based community analysis and automated ATC labeling with literature and docking analysis, narrowing the search space for *in silico* and experimental follow-up. The chloramphenicol example illustrates its utility for uncovering non-obvious repositioning opportunities. Future work will extend similarity definitions (e.g., to higher-order network motifs) and incorporate wet-lab validation of top candidates.

## 1 Introduction

Traditional drug design is challenging, expensive, and time-consuming Fetro and Scherman (2020). In this context, finding new indications for existing drugs—a process known as drug repositioning or repurposing—is an effective and promising strategy for discovering new therapies for both common and rare diseases Tian et al. (2018); Parvathaneni et al. (2019). Indeed, repositioning is an alternative strategy that enables the reuse of approved active pharmaceutical ingredients, significantly reducing development timelines and costs Pushpakom et al. (2019); it also offers greater safety predictability because it involves drugs with known pharmacokinetic profiles that have already undergone rigorous testing Parvathaneni et al. (2019); Pushpakom et al. (2019); Zhang et al. (2020).

Drug repositioning employs computational and experimental approaches, sometimes in combination, to harness the benefits of both Pushpakom et al. (2019); Morselli Gysi et al. (2021); Ko (2020). Computational models leverage data mining, machine learning, and network analysis to uncover interactions not detected during clinical trials, predict drug safety, and explore relationships between drug data and genomic, transcriptomic, and phenotypic data Pushpakom et al. (2019); Morselli Gysi et al. (2021); Udrescu et al. (2016), (2020). However, extracting relevant and meaningful information becomes difficult due to the exponential growth of biomedical data, requiring advanced algorithms and strong data curation Udrescu et al. (2023). Various effective repositioning methods have emerged by integrating information technology, including molecular modeling and data mining techniques, to address such provocation. In this way, refinements in data processing and computational methods have laid the foundation for structured drug repositioning protocols. For example, Jarada et al. (2020) set up a four-step protocol: selecting the strategy based on available datasets, identifying the appropriate computational method and building the model, validating the model, and delivering drug candidates for repositioning.

Network-based models are an important computational drug repositioning framework that integrates complex system theory, data mining, and machine learning; they represent biological systems as nodes (e.g., drugs, diseases, or proteins) interconnected by edges (e.g., the relationships between them). Network-based methods help uncover new drug targets Luo et al. (2017); Amiri Souri et al. (2022); Pham and Tran (2024) or pharmacological properties Udrescu et al. (2016); Udrescu and Udrescu (2019), thus supporting repositioning opportunities. Furthermore, network-based methods working at the macro-scale can uncover repositioning candidates that micro-scale approaches like molecular docking cannot identify Morselli Gysi et al. (2021). One practical approach, which we also use in this paper, is to employ unsupervised machine learning algorithms in network data representations to identify clusters or communities of nodes; these clusters can be labeled according to relevant properties and then serve as the basis for drug repositioning according to the rationale of guilt by association Udrescu et al. (2016), (2020); Groza et al. (2021).

Molecular docking is an *in silico* approach used to simulate drug-target interactions, predicting how a drug molecule binds to a biological target and calculating the binding affinity. Molecular docking can also identify off-target effects (i.e., unaccounted interactions with target proteins) that may indicate new therapeutic uses of existing drugs. However, molecular docking requires significant computational resources, and applying it on a large scale is cumbersome Bender et al. (2021). Therefore, a more efficient approach is to use other computational techniques (such as machine learning and network analysis) to reduce the vast search space of chemical drug-target interactions by identifying a specific list of selected targets for exploration and analysis *via* molecular docking. In this way, docking studies would provide robust hypotheses for further *in vitro* and *in vivo* experimental testing Udrescu et al. (2020); Alam et al. (2022); Sharma et al. (2021).

Computational pipelines for drug repositioning commonly integrate database aggregation, big-data analytics, machine learning, network analysis, and molecular docking (see our analysis of previous work in Section 2). While state-of-the-art multi-stage approaches are effective at prioritizing candidate drugs, they frequently yield only ranked lists of repositioning hypotheses without identifying the specific target activities or mechanisms of action. Consequently, follow-up validation—such as targeted molecular docking or biochemical assays—remains cumbersome and time-consuming in the absence of hinting specific targets and mechanisms of action.

This paper addresses the issue of delivering a drug repositioning hint list and related data that foster molecular docking analysis. To this end, we implement a fully automated computational drug repositioning pipeline that integrates computational network analysis (i.e., community detection in drug-drug similarity networks), community labeling based on the Anatomical Therapeutic Chemical (ATC) drug categorization system, literature-based validation, and ATC level 4 drug-target interaction information, to generate drug repositioning hints along with the list of relevant targets to be further investigated by molecular docking.

Our work proposes the following original contributions to attain the above-stated objectives:

• A drug-drug similarity network for drug repositioning built by projecting a tripartite drug-gene-disease network (with data from DrugBank Wishart et al. (2018) and DisGeNET Piñero et al. (2020));

• The fully automated computational drug repositioning pipeline that integrates drug-drug similarity network clustering, ATC cluster/community labeling, and literature validation of drug repositioning hints (showing an accuracy of 73.6%);

• A methodology that uses level 4 ATC data to indicate suitable targets for drug repositioning validation with molecular docking;

• The illustration of our method’s advantages by performing molecular docking for the chloramphenicol repositioning in cancers driven by Bruton’s tyrosine kinase 1 (BTK1) and the phosphoinositide 3-kinase (PI3K) alpha, gamma, and delta isoforms; this case was predicted by our pipeline.

The remainder of this paper is organized as follows. Section 2 presents the state-of-the-art in computer-automated pipelines for drug repositioning, Section 3 describes our repositioning pipeline, Section 4 presents the pipeline results, Section 5 shows how the results of our pipeline foster molecular docking analysis in the case of chloramphenicol repositioning in cancers, and Section 6 discusses the relevant results and draws conclusions.

## 2 Computational pipelines for drug repositioning hints

Since our paper proposes an automated pipeline for computational drug repositioning, this section provides a comparative overview of computational pipelines that predict new pharmacological properties of drugs and generate lists of candidate drugs for repositioning. Consequently, Table 1 summarizes 20 articles (published between 2013 and 2024) that describe drug repositioning pipelines; for each pipeline, the table presents the computational method, its focus, source and data integration, pipeline automation/validation, and the most relevant outcome.

**TABLE 1:** Comparative overview of computational drug repurposing pipelines. We summarize 20 drug repurposing pipelines based on computational methods, their focus (i.e., exhaustiveness or pathology-specific focus), and data integration and source. The Automation/Validation column specifies whether the pipeline is fully automated and includes automated testing/validation. The Main outcome column highlights the most relevant results of each pipeline.

References	Computational method	Focus	Data source, integration	Automation/Validation	Main outcome
CBDDIN Udrescu et al. (2016)	Network-based	Broad	Drug-drug interactions (DrugBank)	Automated/Not integrated	85% accuracy; large repositioning hint list
DDSN Udrescu et al. (2020)	Network-based	Broad	Drug-target interactions (DrugBank)	Automated/Partial	86.51% accuracy; large repositioning hint list
Groza et al. (2021)	Network-based	Broad	Drug-gene interactions (DrugBank)	Automated/Integrated	Large repositioning hint list
SAveRUNNER Fiscon and Paci (2021); Fiscon et al. (2021a), (b); Conte et al. (2022); Paci et al. (2022)	Network-based	Broad Fiscon and Paci (2021), COVID-19 Fiscon et al. (2021b), ALS Fiscon et al. (2021a), breast cancer Conte et al. (2022), CVD Paci et al. (2022)	Drug-target interactions, Drug-disease associations (DrugBank, CMAP, GEO)	Automated/Integrated	73% accuracy Fiscon and Paci (2021); identified 121/403 hints for ALS Fiscon et al. (2021a); candidate list for breast cancer Conte et al. (2022) and CVD Paci et al. (2022)
Vir2Drug Minadakis et al. (2023)	Network-based	Pathogens	Public pathogen databases	Automated/Not integrated	Flexible drug repositioning tool for pathogens
Morselli Gysi et al. (2021)	Network-based	COVID-19	Public drug and target databases	Automated/Integrated	62% success rate in human cells, 4 drugs repurposable for COVID-19
CANDO Mangione et al. (2020), Mangione et al. (2022)	ML (SVM, RF, LR), Docking	Broad Mangione et al. (2020), COVID-19 Mangione et al. (2022)	Drug-protein, drug-disease relationships (PDB, CTD, DrugBank)	Automated/Integrated	51/275 drugs confirmed anti-SARS-CoV-2 activity Mangione et al. (2022)
SperoPredictor Ahmed et al. (2022)	ML (RF, Tree, GBT)	Broad	Drug-disease data (DrugBank)	Automated/Integrated	> 99% accuracy, tested on COVID-19: literature validation 12/25 drugs
Tuerkova and Zdrazil (2020)	Substructure search, Hierarchical clustering	Broad	Drug-target data (DrugBank, CAS, PDB)	Partial/Integrated	Educational tool validated for GLUT-1 deficiency and COVID-19
Lv et al. (2024)	DL (Transformer)	Epilepsy	Drug-Target Interactions	Automated/Integrated	Lomitapide validated as an anti-seizure candidate
Liu et al. (2021)	Causal inference, DL	Broad	Emulated clinical trials	Automated/Integrated	Tested on coronary artery disease (CAD): identified and ranked 55 potential drugs
ksRepo Brown et al. (2016); Traylor et al. (2021)	Statistical analysis	Broad Brown et al. (2016), Chordoma Traylor et al. (2021)	Omics (GEO, CTD)	Automated/Integrated	Tested on prostate cancer: Identified 5 drugs from > 7,000 compounds Brown et al. (2016); Identified 6/13 candidates Traylor et al. (2021)
DvD Pacini et al. (2013)	Correlation Analysis	Broad	GEO, CMAP, ArrayExpress	Automated/Not integrated	Predicts repositioning candidates and side effects
Shuey et al. (2023)	GWAS mapping	Diabetes	GWAS, EHR	Partial/Integrated	20/283 drugs evidenced for glycemic control

Abbreviations: CBDDIN, Community-Based Drug-Drug Interaction Network; DDSN, Drug-Drug Similarity Network; SAveRUNNER, Searching off-lAbel dRUg aNd NEtwoRk); CANDO, Computational Analysis of Novel Drug Opportunities; ksRepo–Kolmogorov-Smirnov repositioning; DvD–Drug *versus* Disease.

All pipelines presented in Table 1 have three essential components: data retrieval, data analysis/processing and inference, and validation. The data retrieved are integrated into specific structures and fed to the processing and inference stage. The inference component then produces a tentative drug repositioning list to be tested by the validation component; the validation outputs the final repositioning hint list.

The pipelines integrate data from large public datasets and are automated, although some do not integrate validation (see column Automation/Validation in Table 1). In addition, most pipelines are designed for a broad range of repositionings, while some are focused on specific diseases. However, the defining element of drug repositioning pipelines is the computational method for data processing and inference. In Table 1, we have pipelines based on computational network analysis Udrescu et al. (2016), Groza et al. (2021), Fiscon and Paci (2021), Fiscon et al. (2021a), (b), Conte et al. (2022), Paci et al. (2022), Minadakis et al. (2023), Udrescu and Sbarcea (2020), machine and deep learning Morselli Gysi et al. (2021); Mangione et al. (2020), (2022), Ahmed et al. (2022), Tuerkova and Zdrazil (2020), Lv et al. (2024), Liu et al. (2021), and expression-based and statistical approaches Brown et al. (2016), Traylor et al. (2021), Pacini et al. (2013), Shuey et al. (2023), Amadori et al. (2023).

Although, as mentioned, some existing pipelines filter their initial drug repositioning hint list by performing automated validation (with literature records or other tools), none integrate molecular docking analysis because that would entail substantial computational resources. Moreover, no existing pipeline facilitates validation with molecular docking by providing additional information, such as a list of potentially relevant targets for which the drug binding mode can be predicted and its binding affinity estimated.

## 3 Proposed drug-repositioning pipeline

To foster the integration of molecular docking into the analysis and validation of drug repositioning hint lists, we propose a pipeline that has the following components: data retrieval and integration from DrugBank and DisGeNET, inference using computational network analysis (i.e., cluster/community detection), and drug repositioning hint validation by checking literature databases.

**FIGURE 1:**
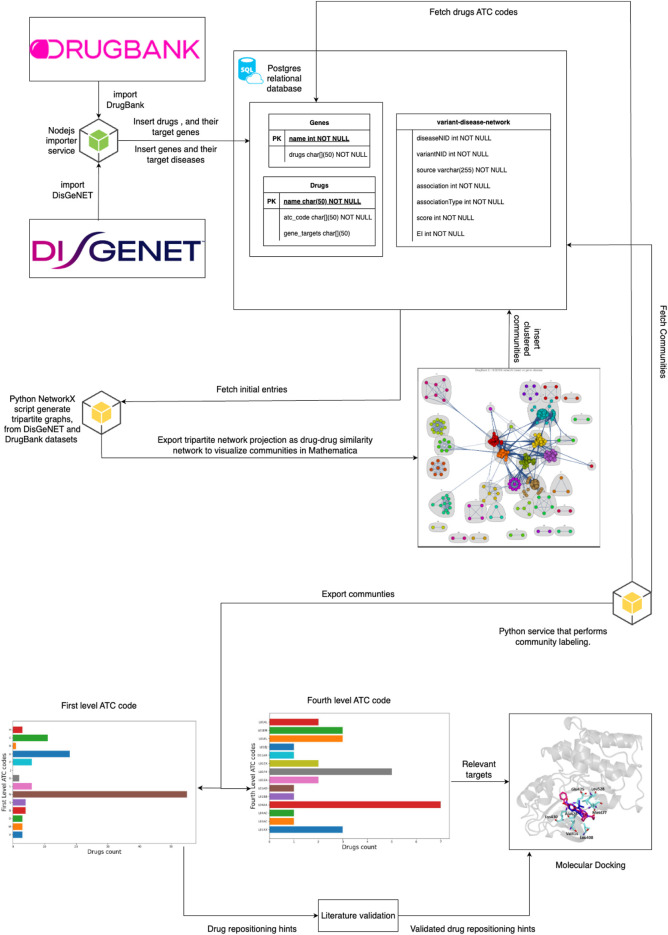
The overview of our proposed drug repositioning pipeline. We present the pipeline components as the information flows from the data sources (DrugBank and DisGeNET) to the repositioning hint list. The retrieved data is integrated into the Postgres Relational Database (PDR); then, we query the database to build a tripartite drug-gene-disease graph and project the tripartite graph as a weighted drug-drug similarity network (a bigger edge weight means a higher similarity). We perform clustering (i.e., community detection) on the drug-drug similarity network and store the cluster/community structure in the PDR. After an automated analysis of level 1 ATC code distribution in the resulting communities, we assign the dominant label to each cluster; we also perform a similar distribution analysis for level 4 ATC codes, which identifies the relevant targets for the potential repositionings. The ATC code distribution analysis and labeling results are stored in the PDR. Drugs that do not match their cluster label (based on existing DrugBank information) are added to the initial drug repositioning hint list, which is then refined through the Literature Validation component. Finally, the validated hints and the lists of relevant targets are delivered to Molecular Docking testing.

Figure 1 provides the overview of our project by indicating the information flow from the data sources (drug-gene interactions from DrugBank and DisGeNET gene-disease associations), going through data processing and inference through tripartite network projection and network community/cluster detection, then cluster labeling with level-1 ATC codes to produce the initial repositioning hint list and finding relevant targets using level 4 ATC codes, and finally to the literature-based testing that produces the validated drug repositioning hint list. In this way, for each hint validated by the literature search, we have an additional list of potentially relevant targets that can be investigated through molecular docking. Indeed, having information on the anatomical groups targeted by the repositionings and the relevant targets is a significant advantage for the docking process because it substantially narrows its search space. An additional component in our pipeline implementation is the Postgres Relational Database, which integrates all data retrieved from DrugBank and DisGeNET with data generated from community detection and ATC codes.

The following subsections describe the main features of our pipeline presented in Figure 1. We provide the pipeline implementation in our GitHub repository.

### 3.1 Drug-drug similarity network (DDSN)

The component dedicated to data retrieval and integration extracts information on drug-drug similarity relationships. As such, it builds a drug-drug similarity network based on indirect drug-disease relationships, using data from DrugBank Wishart et al. (2018) and DisGeNet Piñero et al. (2020). Indeed, neither database provides direct connections between drugs and diseases; therefore, we first combined DrugBank (containing data on drug-gene interactions) and DisGeNet (containing data on gene-disease relationships) to create a drug-gene-disease tripartite network. We used a Node. js script to import and process the data. Then we build the tripartite network as a graph $G=\left(V,E\right)$ , where V represents the set of vertices or nodes, and E the set of edges or links between the nodes. In our tripartite network, set V is the union of the three disjoint sets of vertices represented by drugs/medicines $\left(V_{m}\right)$ , genes $\left(V_{g}\right)$ , and diseases $\left(V_{d}\right)$ , with $V=V_{m}\cup V_{g}\cup V_{d}$ . The set of edges E is the union of the disjoint sets of drug-gene edges $\left(E_{g}\right)$ and gene-disease edges $\left(E_{d}\right)$ , $E=E_{g}\cup E_{d}$ . The directed edges $e_{ij}^{g}\in E_{g}$ represent the interaction between $v_{i}^{m}\in V_{m}$ and $v_{j}^{g}\in V_{g}$ (i.e., drugs and genes), and the directed edges $e_{ij}^{d}\in E_{d}$ represent the interaction between $v_{i}^{g}\in V_{g}$ and $v_{j}^{d}\in V_{d}$ (i.e., genes and diseases).

Next, we projected the tripartite network into a monopartite drug-drug similarity network $G_{s}=\left(V_{m},E_{s}\right)$ , where the vertices are medicines $\left(V_{m}\right)$ linked by similarity edges $\left(E_{s}\right)$ . A similarity undirected edge $e_{ij}^{s}\in E_{s}$ between $v_{i}^{m}$ and $v_{j}^{m}\in V_{m}$ exists if there is at least one path between a drug/medicine $v_{i}^{m}$ and a disease $v_{k}^{d}\in V_{d}$ and at least one path between $v_{j}^{m}$ and the same disease $v_{k}^{d}$ in G . The similarity network $G_{s}$ is weighted, with the weight of $e_{ij}^{s}$ representing the number of w diseases $v_{k}^{d}\in V_{d}$ that are connected (*via* a path) to both $v_{i}^{m}$ and $v_{j}^{m}$ in the tripartite graph G . A connecting path between $v_{i}^{m}\in V_{m}$ and $v_{j}^{d}\in V_{d}$ exists in the tripartite graph G if we have a gene $v_{k}^{g}\in V_{g}$ such that ∃ two directed edges, $e_{ik}^{g}\in E_{g}$ from $v_{i}^{m}$ to $v_{k}^{g}\in V_{g}$ and $e_{kj}^{d}\in E_{d}$ from $v_{k}^{g}$ to $v_{j}^{d}\in V_{d}$ . According to the formal description above, the weight of a link/edge in the DDSN $G_{s}$ (representing the similarity relationship between two drugs/medicines) is the number of diseases that can be reached *via* valid connecting paths in the tripartite graph from both drugs.

**FIGURE 2:**
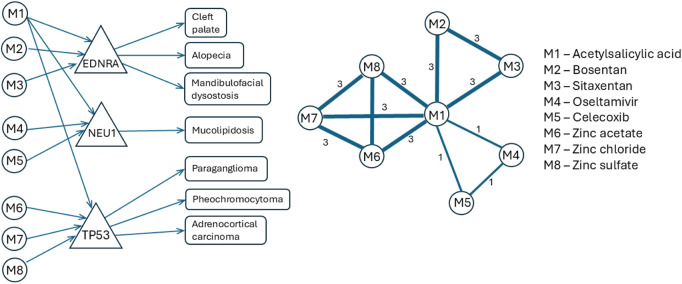
An example of how our algorithm projects the tripartite (drug-gene-disease) network onto a weighted drug-drug similarity network. The left shows the tripartite graph G built with information retrieved from DrugBank and DisGeNET: the circle nodes represent drugs in $V_{m}$ , the triangle nodes represent genes in $V_{g}$ , the box nodes represent diseases in $V_{d}$ , and the directed edges (i.e., arrows) represent drug-gene interactions from $E_{g}$ and gene-disease associations from $E_{d}$ . The center depicts the weighted drug-drug similarity network $G_{s}$ , where the circles represent drugs and the undirected edges represent similarity relationships $E_{s}$ —a thicker edge (according to its weight) means a stronger similarity (For instance, we have an edge of weight 3 between M1 and M7 because we have valid paths connecting 3 diseases to both drugs: Paraganglioma, Pheochromocytoma, and Adrenocortical carcinoma.) The right shows the drug names.

Figure 2 presents an example of tripartite (drug-gene-disease) graph projection onto a drug-drug similarity network according to the algorithm described in this section; the example uses a small subgraph extracted from G .

### 3.2 Network community detection, labeling, and target identification

Network community detection (also known as graph clustering) identifies groups of nodes/vertices that we call communities or clusters ( $C_{1},C_{2},\ldots C_{n}$ , with $C_{1}\cup C_{2}\cup \ldots \cup C_{n}=V_{m}$ ), which are more densely connected internally than the rest of the graph. We perform network community detection in Wolfram Mathematica 13 Inc. (2023) using the FindGraphCommunities function with the ‘Hierarchical’ method, based on vertex similarity and a dendrogram to represent nested community structures.

For each cluster $C_{i}$ $\left(i=\bar{1.n}\right)$ , we automatically assign a cluster’s dominant level 1 ATC code as its label and add drugs deviating from the cluster label to the drug repositioning hint list. We do this by computing cluster $C_{i}$ ’s ATC code histogram $k^{i}=\left(k_{1}^{i},k_{2}^{i},\ldots k_{W}^{i}\right)$ (where W is the number of distinct ATC codes in $C_{i}$ and $k_{j}^{i}$ the number of drugs in $C_{i}$ that have ATC code j ); as such, the dominant ATC code is j if $\max\left(k_{1}^{i},k_{2}^{i},\ldots k_{W}^{i}\right)=k_{j}^{i}$ . As a result, we add all drugs $v\in C_{i}$ that do not have the dominant ATC level 1 code j (according to DrugBank) to the repositioning list as potentially having the property described by j .

DrugBank lists all associated level 4 ATC codes for each target; therefore, if we establish the dominant level 4 ATC codes in each cluster, we can identify the cluster’s targets of interest, which will subsequently foster the molecular docking validation efforts. Accordingly, for all drugs within the cluster labeled with the dominant level 1 ATC, we systematically compute the histograms corresponding to their levels 2, 3, and 4 of ATC and also identify the dominant level 4 ATC codes in each cluster, map them with their associated biological targets from DrugBank, and associate these targets with drug repositioning hints. Specifically, if the dominant ATC level 4 code in $C_{i}$ corresponds to the mechanism of action a , then we retrieve from DrugBank the list of targets associated with that mechanism of action $T_{a}$ .

Given that we can assemble drugs according to a specific mechanism of action (described by a level 4 ATC code) and its corresponding biological targets, our approach uses shared pharmacological properties to suggest new therapeutic uses. For example, a drug not known to have the community-dominant ATC level 4 code could be uncovered as binding to a target shared by the dominant group; this might be effective for a disease treated by the community’s dominant drug class.

The targets we identify to associate with the drug repositioning hints facilitate molecular docking (by calculating their binding affinity to the assigned targets), thus prioritizing drugs with favorable interaction profiles. This strategy improves the efficiency of subsequent experimental validation by focusing on biologically plausible hypotheses.

### 3.3 Validation with literature database search

We validate repositioning candidates by automatically querying the PubMed database with Biopython Cock et al. (2009). Explicitly, we assign the Medical Subject Headings (MeSH) terms corresponding to each drug name in the repositioning candidate list, its community level 1 ATC name, and relevant synonyms. We used the Boolean operator ‘AND’ to correlate the drug name and the ATC category name, and ‘OR’ to include relevant synonyms of the ATC category. To rely only on high-quality evidence, we applied filters that retrieve only research articles and clinical trials, excluding commentaries and unrelated studies. Subsequently, we pass the retrieved literature list through expert analysis that filters the publications and further confirms the predicted property.

Validation of drug repositioning hints with the latest literature and electronic health databases is paramount. The main problem in drug repositioning is that we do not have a robust ground truth: we can rely on what we know about drugs (i.e., current knowledge), but we cannot rely on negative information. In other words, if we do not know, for instance, that there is a specific drug-target interaction, it does not necessarily mean that the interaction does not exist; maybe it exists, but we do not know about it yet Udrescu et al. (2023). Therefore, most available drug repositioning pipelines based on machine learning methods adopt the train/test dataset split strategy to assess their performance. However, such a strategy cannot work well in the case of network-based approaches, as it entails affecting the network topology; this is why network-based methods rely on external validation for performance assessment. External validation can be performed by automatic/systematic literature or electronic health databases, molecular docking, *in vitro*, or *in vivo* actual experiments. Unfortunately, *in vitro* and *in vivo* experiments require extensive resources and demand carefully designed protocols, meaning serious additional research. On the other hand, molecular docking requires substantial computational resources, including months of program execution run time. Therefore, validation of drug repositioning hints with the latest literature and electronic health databases remains the most affordable and feasible solution (adopted by most state-of-the-arthe weight of the similarity relationship between two drugs/medicines is the number of diseases that can be reached *via* valid paths in the tripartite graph from both drugs t pipelines in Table 2).

**TABLE 2:** Features of the clusters in our drug similarity network generated by the hierarchical clustering algorithm. For each cluster, we provide the number of component drugs and the corresponding label corresponding to the level 1 ATC of the majority of drugs in the first and second columns. The column *% Predominant ATC in DB* lists the percentages of drugs whose level 1 ATC listed by DrugBank predominates in each cluster. The column *% Confirmed by literature* presents the percentages of drugs for which the literature confirms the property represented by the level 1 ATC, thus increasing the prediction accuracy of our drug-drug similarity network, as presented in column *% Accuracy*.

Cluster	No	ATC level 1	% predominant ATC in DB	% confirmed by literature	% accuracy
1	105	N (Nervous system)	52.4	26.7	79.0
2	88	B (Blood and blood forming organs)	52.3	14.8	67.0
3	95	C (Cardiovascular system)	35.8	13.7	49.5
4	85	L (Antineoplastic and immunomodulating agents)	52.9	30.6	83.5
5	51	D (Dermatologicals)	54.9	17.6	72.5
6	51	L (Antineoplastic and immunomodulating agents)	60.8	23.5	84.3
7	51	M (Musculo-skeletal system)	64.7	15.7	80.4
8	44	G (Genito-urinary system and sex hormones)	61.4	20.4	81.8
9	31	N (Nervous system)	61.3	16.1	77.4
10	24	L (Antineoplastic and immunomodulating agents)	87.5	4.2	91.7
11	15	H (Systemic hormonal preparations)	40	13.3	53.3
12	13	D (Dermatologicals)	30.8	46.1	76.9
		Total	53.4	20.2	73.6

## 4 Pipeline results

This section presents the results that we obtained by applying our proposed pipeline to the DrugBank 5.1.9 and DisGeNET data. In particular, we extracted information on drug-gene edges $E_{g}$ from DrugBank and on gene-disease edges $E_{d}$ from DisGeNET.

### 4.1 Network analysis

**FIGURE 3:**
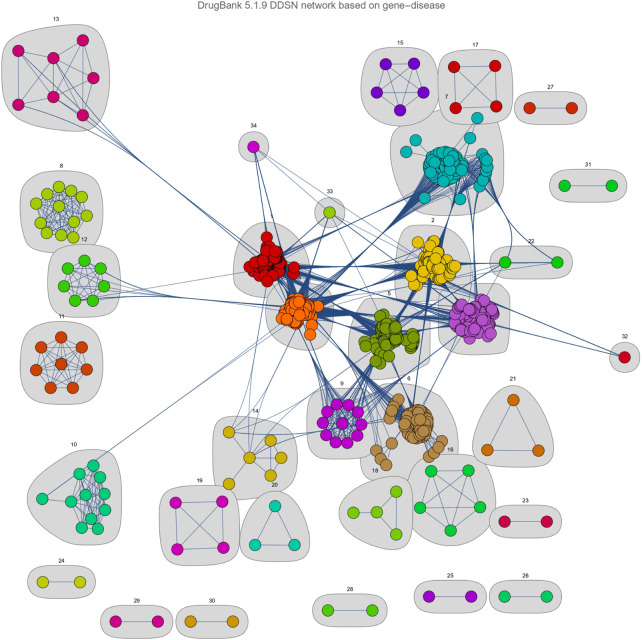
The clustered Drug-Drug Similarity Network (DDSN). We present the graphical representation of communities/clusters $C_{1},C_{2},\ldots C_{n}$ (n=34) generated for network $G_{s}$ , where nodes represent drug/medicines and weighted links represent similarity relationships (a higher weight corresponds to a stronger similarity). The graphical representation assigns distinct colors to nodes in each cluster, highlights clusters with gray background, and assigns cluster numbers according to size (i.e., number of nodes in the cluster).

Figure 3, shows that our DDSN network-building and clustering methodology produces 34 clusters. However, we excluded from further analysis all clusters disconnected from the main connected component (clusters 15–21 and 23–31) and clusters with fewer than eight vertices/drugs (clusters 13, 14, 22, 32–34). Consequently, we investigate the remaining 12 clusters in the drug-drug similarity network (Figure 3) as follows:

1. Scan the level 1 ATC of all drugs in DrugBank and automatically label the cluster with its predominant level 1 ATC property (see the first four columns in Table 2).
2. Automatically inspect the literature for drugs with ATC level 1 that differs from those representing the cluster label; we selected articles reporting the pharmacological property of interest, thus confirming the ATC-based community, as presented in Table 2, columns *% Confirmed by literature* and *% Accuracy*, respectively.
3. Add the nodes for which the literature has not confirmed the cluster’s pharmacological property to the list of drug candidates for repositioning (Table 2, column *% Repositioning candidates*).

Our results show that 53.4% of drugs have the ATC level 1 property given by their cluster label, and 20.2% are not formally classified in the ATC level 1 cluster label with DrugBank data Wishart et al. (2018), but the automated literature check demonstrates the predicted corresponding anatomical/pharmacological properties (see our GitHub main results). Consequently, 26.4% of the drugs do not comply with the cluster label, so we consider them candidates for repositioning on the property corresponding to their community/cluster label (see Table 2). In addition, this means that the accuracy of our pipeline, measured with the available information (that is, current knowledge), is 53.4%+20.2%=73.6% .

### 4.2 ATC analysis and inference

**FIGURE 4:**
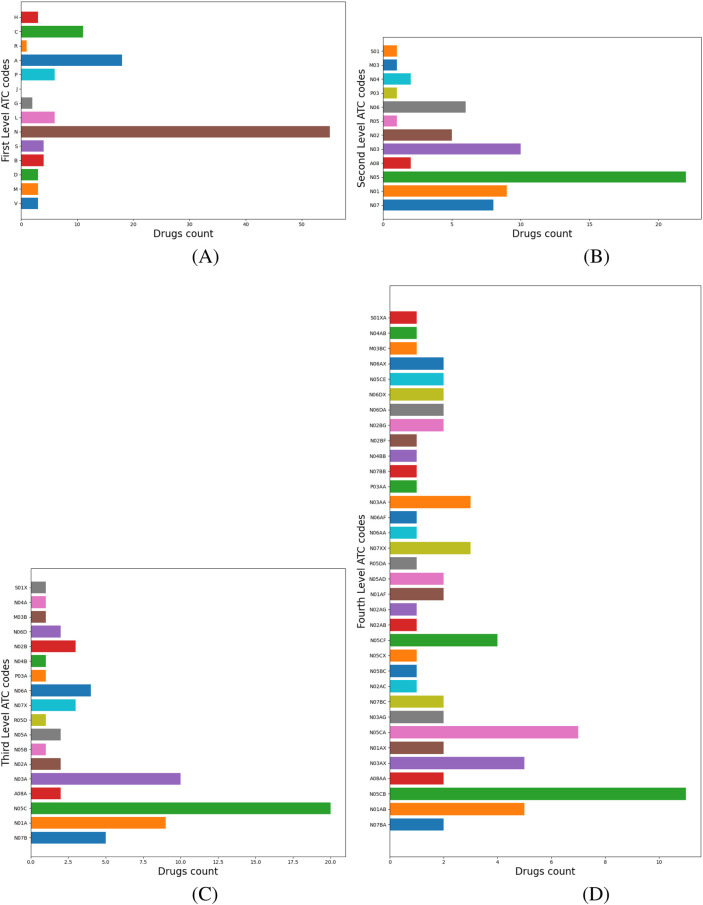
Cluster 1 histograms for ATC levels 1–4. **(A)** Drugs with level-1 ATC code N—*Nervous System* dominate, hence the cluster label. **(B)** Distribution of N-classified drugs across ATC level 2 codes. **(C)** Distribution across ATC level 3 codes. **(D)** Distribution across ATC level 4 codes.

ATC level 1 codes describe the main anatomical or pharmacological drug groups. ATC level 4 codes correspond to the chemical, pharmacological, or therapeutic drug subgroups. Our method identifies the dominant ATC level 1 and level 4 codes of the cluster and proposes them as new anatomical and physiological target groups and potential mechanisms of action for repositioning candidates. Figure 4 illustrates the labeling process for Cluster 1, where the dominant level 1 ATC code is N (Nervous System); therefore, N is automatically assigned as the cluster’s label, as shown in panel (a). Panel (b) presents a histogram of drugs distributed across their level 2 ATC codes, with N05–*Psycholeptics* as the dominant category. Panel (c) further refines this distribution at level 3 ATC, showing that the majority of drugs fall under N05C–*Hypnotics and Sedatives* and N03–*Antiepileptics*. Analysis of level 4 ATC codes reveals the top three categories, N05CB and N05CA (barbiturates), and N03AX (Other Antiepileptics), depicted in panel (d). We extract all nervous system-related targets associated with N05CB, N05CA, and N03AX drugs from DrugBank and propose their evaluation *via* molecular docking to identify potential candidates for repositioning within Cluster 1.

Our pipeline generates a list of drug candidates for repositioning at varying first ATC levels. The project README in our GitHub (https://github.com/GrozaVlad/Drug-repurposing-using-DDSN-with-disgenet/blob/main/results/README.md) presents two result files: Literature validation. xlsx, which provides the PubMed link(s) and year of publication for literature supporting the predicted properties, and Repositioning hints and predicted targets. xlsx, which lists the pipeline’s the top 3 ATC level 4 codes (ranked by frequency within the community) and the DrugBank targets associated with drugs in those ATC level 4 groups.

Given the challenge of finding anticancer therapies, we selected an old drug from an L-labeled community to further validate our method *in silico* with molecular docking. To this end, we selected chloramphenicol, which has been in clinical practice for decades; its pharmacokinetics, safety profile, and side effects are well understood, which can expedite its repositioning as a cancer therapeutic. In addition, chloramphenicol is inexpensive, making it an attractive option for cancer treatment from economic considerations. The existing approval for chloramphenicol as an antibacterial agent and its age could simplify the regulatory pathway for repositioning in cancer and may lead to faster clinical trials and approval.

**FIGURE 5:**
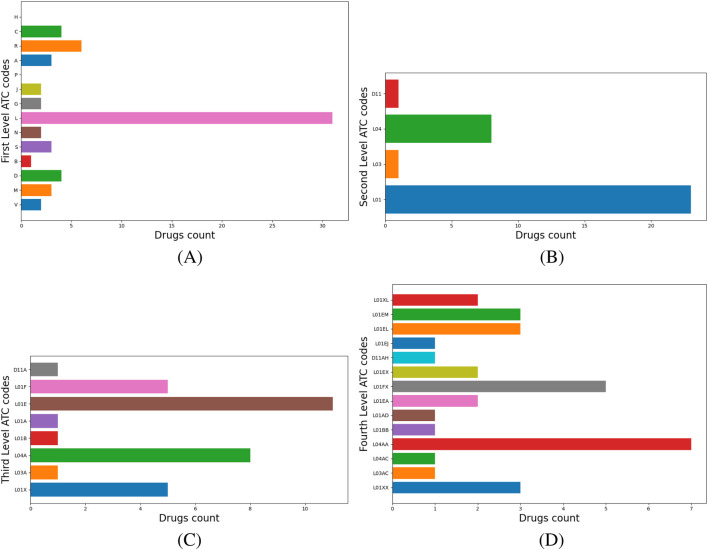
Cluster 6 histograms levels 1 to 4. **(A)** Drugs with level 1 ATC codes L–*Antineoplastic and immunomodulating agents* dominate; consequently, this code becomes the cluster’s label .**(B)** The distribution of L-classified drugs across their respective level 2 ATC codes. **(C)** The distribution of L-classified drugs across their respective level 3 ATC codes. **(D)** the distribution of L-classified drugs across their respective level 4 ATC codes.

Chloramphenicol belongs to Cluster 6, where 60.8% of drugs have ATC level 1 code L–*Antineoplastic and immunomodulating agents* (see Figure 5A), so we add chloramphenicol to the drug repositioning hint list as an anticancer drug. Inspection of the higher ATC levels of L drugs reveals their distribution across ATC levels 2 as follows: 23 drugs have code L01–*Antineoplastic agents* (e.g., pentostatin, dasatinib, axicabtagene ciloleucel), eight drugs have L04–*Immunosuppressants* (e.g., cladribine, tofacitinib, abatacept), and one has L03–*Immunostimulants* (namely, aldesleukin) (Figure 5B). Next, the ATC level 3 distribution analysis shows that sublevel L01E–*Protein kinase inhibitors* dominates with 11 drugs, followed by L04A–*Immunosuppressants* with eight drugs (Figure 5C). Figure 5D illustrates the distribution of drugs on ATC level 4: eight drugs have code L04AA–*Selective immunosuppressants*, five drugs each have L01FX–*Other monoclonal antibodies and antibody drug conjugates* and L01XX–*Other antineoplastic agents*, respectively, and 3 drugs each have L01EM–*Phosphatidylinositol-3-kinase (PI3K) inhibitors* and L01EL–*Bruton’s tyrosine kinase (BTK) inhibitors*, respectively.

According to our repositioning pipeline, we assess the top 3 level 4 ATC codes in Cluster 6 to find the repositioning candidate targets. The seven selective immunosuppressor drugs labeled as L04AA target various proteins, such as the ribonucleoside-diphosphate reductase protein group (e.g., RRM1, RRM2, and RRM2B) and the catalytic subunits of the DNA polymerase (e.g., POLA1, POLE, POLE2, POLE3, and POLE4). Monoclonal antibody drugs within the L01FX subgroup target Fc-gamma I, IIa, III-A, III-B, cytotoxic T-lymphocyte protein 4, and many other specific biological targets. The subgroups L01XX, L01EM, and L01EL are in third place. The L01XX subgroup includes other antineoplastic agents for which DrugBank lists targets, such as adenosine deaminase, B-lymphocyte antigen CD19, interleukin-2 receptor subunits alpha and beta, G1/S-specific cyclin-D1, and transcription factor Jun. L01EM drugs are phosphatidylinositol 3-kinase (PI3Ks) inhibitors, and L01EL are Bruton’s tyrosine kinase (BTK) inhibitors.

## 5 Molecular docking analysis

In this section, we consider PI3K and BTK (identified in section 4.2) as targets for the investigation of chloramphenicol’s anticancer potential with molecular docking due to the smaller number of targets to test and, thus, more reasonable simulation time. We provide all the details to perform these simulations, ensuring the reproducibility and robustness of the results.

Despite its age, the scarcity of chloramphenicol testing in cancer was unexpected. The literature reveals only a few references, which do not specifically present tests for chloramphenicol’s anticancer effect. For example, P.C. Giannopoulou et al. and O.N. Kostopoulou et al. reported the synthesis and evaluation of chloramphenicol derivatives that demonstrated cytotoxicity for ZL34 cancer cells and inhibited the growth of T-leukemic cells without influencing the viability of normal human lymphocytes, respectively Giannopoulou et al. (2019); Kostopoulou et al. (2015). A relationship between chloramphenicol and cancer is the triggering of aplastic anemia and leukemia following systemic administration Yuan and Shi (2008) but not after topical use Smith et al. (2000). Chloramphenicol has limited use as an antibacterial because it suppresses bone marrow function by inhibiting mitochondrial protein synthesis; however, the mechanism of this adverse effect could be capitalized in the treatment of leukemia and multiple myeloma Tian et al. (2016). Furthermode, DrugBank lists no clinical trial for chloramphenicol as a potential anticancer agent.

### 5.1 Molecular docking rationale and method

We employ molecular docking simulations as a computational screening tool to validate the drug repositioning candidates identified from network-based clustering and ATC code labeling. This approach tests the hypotheses generated by our pipeline, which assigns biological targets to drugs with divergent level 4 ATC codes within each cluster, as described in Section 3.2.

**FIGURE 6:**
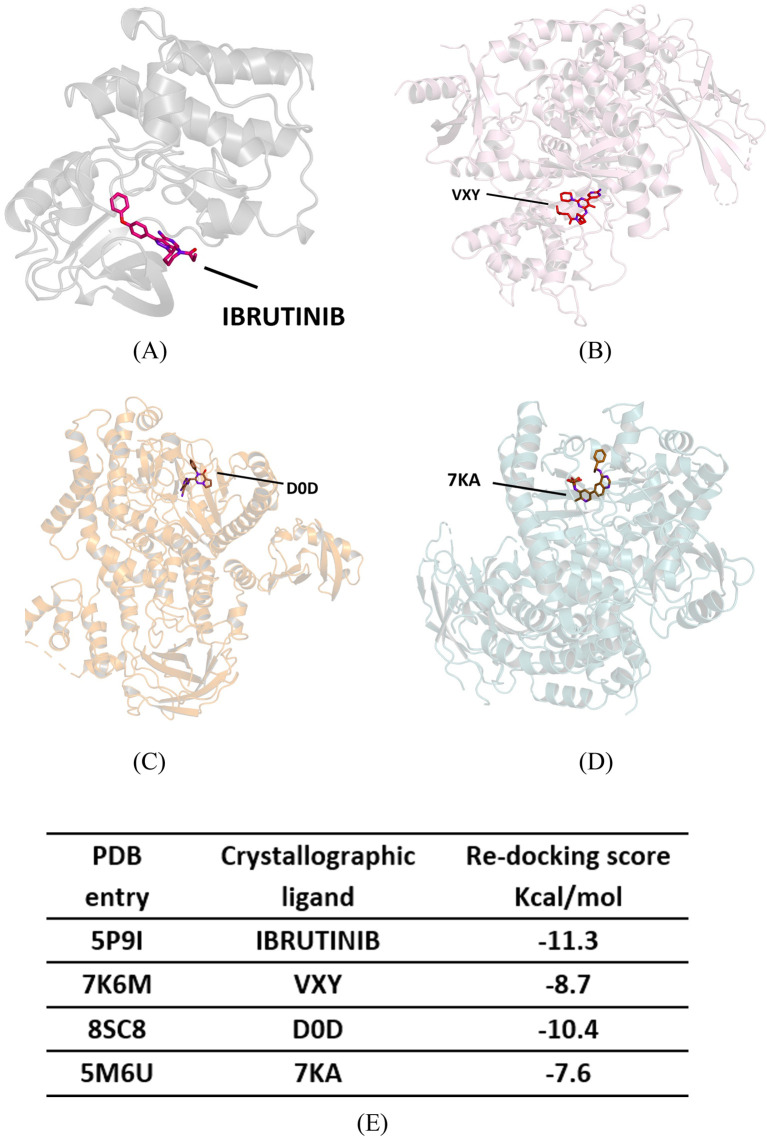
Crystallographic structure of four target proteins studied, along with their corresponding co-crystallized ligands and re-docking scores. **(A)** Crystallographic structure of Bruton’s Tyrosine Kinase (BTK1)—PDB 5P9I. **(B)** Crystallographic structure of the phosphoinositide 3-kinase (PI3K) alpha—PDB 7K6M. **(C)** Crystallographic structure of PI3K gamma—PDB 8SC8. **(D)** Crystallographic structure of PI3K delta—PDB 5M6U. **(E)** Re-docking scores for crystallographic ligands.

**FIGURE 7:**
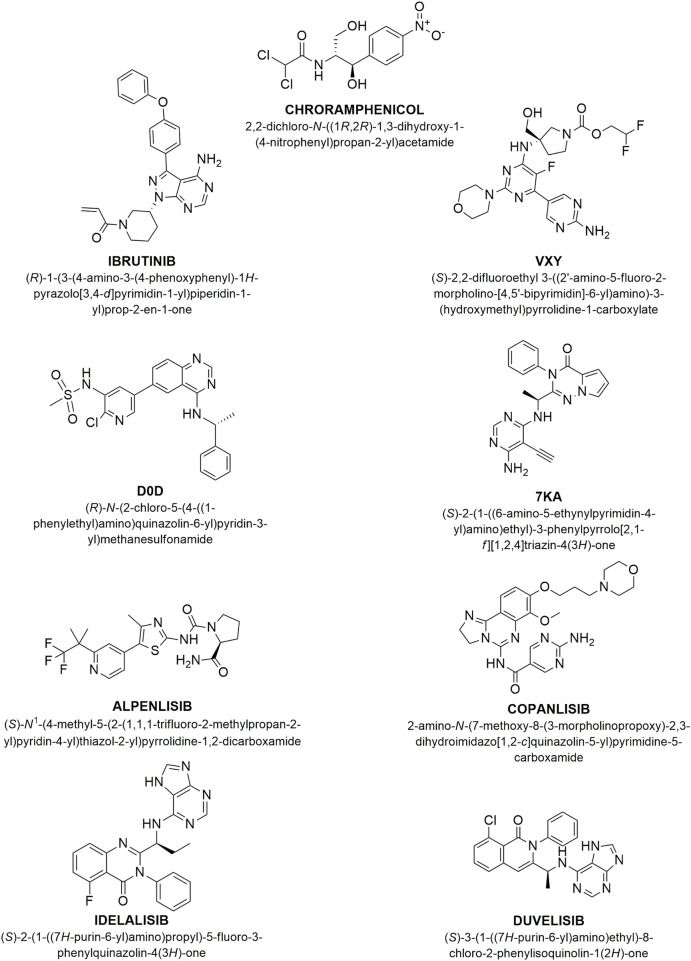
Chemical structure of studied compounds. This figure shows the chemical structures and IUPAC names of the compounds investigated: Ibrutinib, a well-known BTK1 inhibitor; VXY, a synthetic selective morpholine inhibitor targeting the PI3K alpha isoform; D0D, a quinazolinpyridinylmethanesulfonamide inhibitor of PI3K gamma; 7KA, a phenylpyrrolotriazinone inhibitor specific to the PI3K delta isoform; alpelisib, a recognized PI3K alpha inhibitor; copanlisib, an established inhibitor of both PI3K alpha and delta isoforms; idelalisib and duvelisib, both inhibitors of the PI3K gamma and delta isoforms.

We performed molecular docking on the crystallographic structures of Bruton’s tyrosine kinase (BTK1) and the alpha, gamma, and delta isoforms of phosphoinositide 3-kinase (PI3K), all of which belong to *Homo sapiens*. We obtained the crystallographic structures of the target proteins from the Protein Data Bank: PDB codes 5P9I for BTK1 Bender et al. (2017) and 7K6M, 8SC8, and 5M6U for PI3K alpha, gamma, and delta isoforms, respectively (see Figures 6A–D) Cheng et al. (2020); Erra et al. (2017). In 5P9I, BTK1 is co-crystallized with the known inhibitor ibrutinib; the PI3K isoforms in 7K6M, 8SC8, and 5M6U are co-crystallized with synthetic ligands VXY, D0D, and 7KA, respectively (see Figure 7).

We adopted a protein-based approach to study the mode of interactions with the enzyme active site, using a protocolalready adopted in our previous studies Tundis et al. (2023); Perri et al. (2023). As a first step of our *in silico* experiments, a re-docking calculation was performed to determine the binding energy values of the crystallographic ligands for each target protein (see Figure 6E); we used these values as a reference for subsequent simulations.

The molecular structures of alpelisib, copanlisib, idelalisib, duvelisib, and chloramphenicol were built using Avogadro modeling software Hanwell et al. (2012). We employed AutoDock Vina 1.1.2 for docking calculations Trott and Olson (2010). Preliminary conversion of the structures from the PDB format was performed using the AutoDock Tools 1.5.6 graphical user interface Morris et al. (1998). During the conversion, we added polar hydrogens to the crystallographic enzyme structures and merged the ligands’ apolar hydrogens with the carbon atoms to which they are attached. Full flexibility was ensured for the ligands, resulting in four active torsions for duvelisib, five for alpelisib, idelalisib, chloramphenicol, and nine for copanlisib. We conducted all simulations for each compound to a very high degree of exhaustiveness. We analyzed the ligand binding modes through visual inspection and evaluated the intermolecular interactions using the automated protein-ligand interaction profiler, PLIP Salentin et al. (2015).

### 5.2 Molecular docking results

In the modern approach to scientific research, computational techniques provide significant advantages for streamlining drug discovery or uncovering the biological properties of natural or synthetic compounds Grande et al. (2020), (2021). Similarly, these techniques can support the discovery of additional pharmacological activities of known drugs used to treat diseases other than those for which they are currently approved Fadlalla et al. (2022).

The possibility of reusing drugs with already established safety profiles and pharmacokinetics offers the extra advantage of significantly lowering the costs and time required for the standard drug discovery process. To this end, encouraged by the results of previous studies Grande et al. (2020), we conducted molecular docking studies to explore the potential of chloramphenicol to directly interact with Bruton’s tyrosine kinase 1 (BTK1) and the alpha, gamma, and delta isoforms of phosphoinositide 3-kinase (PI3K).

BTK1 is a kinase protein containing five domains: an amino-terminal pleckstrin homology (PH) domain, a proline-rich TEC homology (TH) domain, SRC homology (SH) domains SH2 and SH3, and a protein kinase domain endowed with tyrosine phosphorylation activity Pal Singh et al. (2018).

**FIGURE 8:**
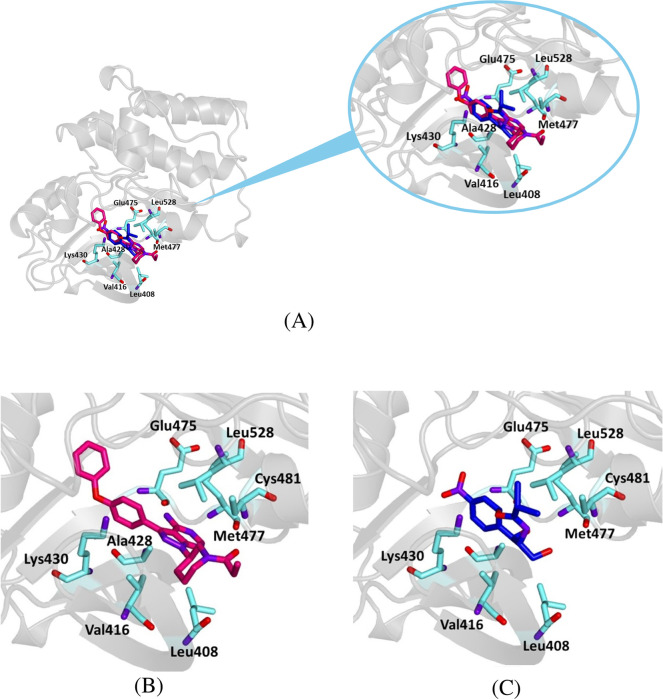
Crystallographic structure of Bruton’s tyrosine kinase (BTK1) corresponding to the PDB entry 5P9I. The protein backbone is represented in the background as ribbons and key amino acid residues of the catalytic site are in cyan. **(A)** Superimposed binding modes of the crystallographic ligand ibrutinib (dark pink) and chloramphenicol (blue). **(B)** The specific binding modes of ibrutinib. **(C)** The specific binding modes of chloramphenicol.

As a result, even though the chloramphenicol–5P9I estimated binding energy ( −7.5 kcal/mol) is lower than that obtained for the crystallographic ligand, it is still a value compatible with a stable complex. Furthermore, chloramphenicol accommodates into the protein binding site, occupying the same position as ibrutinib (Figure 8). The ligand-target complex is stabilized by a hydrogen bond with Lys 430 and hydrophobic and van der Waals interactions with other key residues of the active site (Table 3).

**TABLE 3:** Binding energy values for ligands complexed with the BTK1 catalytic subunit and key protein residues interacting with the ligands.

Ligand	Binding energy [Kcal/mol]	Interactions					
		Hydrogen bonds				Hydrophobic interactions residues	π stacking
		Residues	Distance (Å)		Donar angle [°]		
			H–A	D–A			
Ibrutinib	− 11.3	Glu 475	1.95	2.86	152.53	Val 416	Phe 540
		Met 477	2.05	2.99	158.41	Ala 428	
		Cys 481	1.86	2.82	165.66	Lys 430	
						Leu 528	
Chloramphenicol	− 7.5	Lys 430	2.42	3.09	122.65	Val 416	Lys 430
						Ala 428	
						Lys 430	
						Thr 474	

Class I PI3K includes two subclasses: IA (PI3K alpha, beta, delta) and IB (PI3K gamma). All of them function as heterodimers, consisting of a catalytic subunit (p110) and a regulatory subunit (p85 for subclass IA and p84/p87 for subclass IB, respectively) Vanhaesebroeck et al. (2010). Previous studies using multiple sequence alignment of protein sequences from available crystallographic structures of PI3K identified key residues in the binding regions of each isoform. For PI3K alpha, the critical residues include Ser 774, Trp 780, Asp 810, Tyr 836, Val 851, and Asp 933. Other residues, such as Ser 773, Asn 853, Ser 854, His 855, and Gln 859, appear important for ligand binding. For PI3K gamma, the key residues include Val 882, Asp 964, Tyr 867, Ser 806, and Lys 833. In the case of PI3K delta, Val 828, Trp 760, Lys 779, Glu 826, and Tyr 813 are significant for ligand binding. Identifying compounds interacting with these residues may help develop selective inhibitors for each PI3K isoform Al Hasan et al. (2023).

Considering these details and our preliminary results, we performed molecular docking experiments on the alpha, gamma, and delta isoforms of PI3K to compare the interaction mode of known ligands with that of chloramphenicol. Specifically, to investigate the interaction of chloramphenicol with the PI3K alpha isoform, we used the structure retrieved from PDB with code 7K6M, which corresponds to the enzyme’s catalytic subunit. In this structure, the protein is co-crystallized with a selective morpholine inhibitor, (*S*)-2,2-difluoroethyl-3-((2′-amino-5-fluoro-2-morpholino-[4,5′-bipyrimidin]-6-yl)amino)-3-(hydroxymethyl)pyrrolidine-1-carboxylate, referred to as VXY, discovered through structure-based drug design (SBDD) and computational analysis Cheng et al. (2020).

**FIGURE 9:**
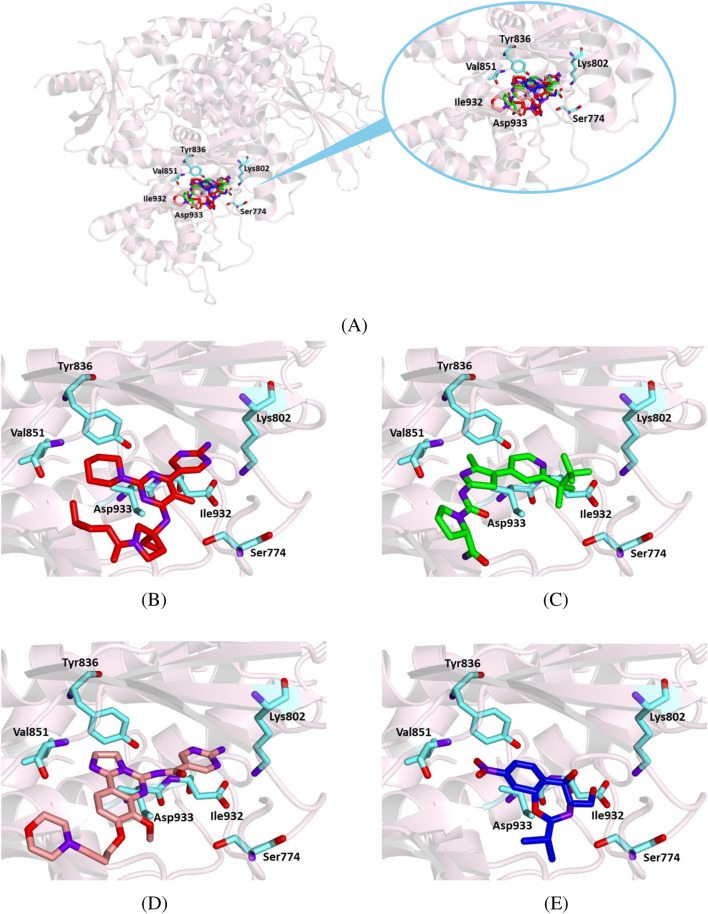
Crystallographic structure of PI3K alpha corresponding to the PDB entry 7K6M. The protein backbone is represented in the background as ribbons and key amino acid residues of the catalytic site are in cyan. **(A)** Superimposed binding modes of the crystallographic ligand VXY (red), alpelisib (green), copanlisib (salmon), and chloramphenicol (blue). **(B)** The specific binding mode of VXY. **(C)** The specific binding mode of alpelisib. **(D)** The specific binding mode of copanlisib. **(E)** The specific binding mode of chloramphenicol.

For a more comprehensive understanding of chloramphenicol’s behavior in its interaction with the target protein, we aimed to compare its binding mode with those of known ligands, such as alpelisib and copanlisib. Accordingly, we docked all compounds with 7K6M. As a result, chloramphenicol shares a similar orientation within the active site as the crystallographic ligands, alpelisib and copanlisib (Figure 9), interacting with Ser 774 throughout a hydrogen bond and with other key binding site residues through hydrophobic and van der Waals interactions (Table 4). The calculated binding energy for the chloramphenicol–7K6M complex, although less favorable than those observed for known ligands, seems to support our hypothesis of a direct interaction between chloramphenicol and PI3K alpha.

**TABLE 4:** Binding energy values for ligands complexed with the PI3K alpha catalytic subunit and key protein residues interacting with the ligands.

Ligand	Binding energy [Kcal/mol]	Interactions					
		Hydrogen bonds				Hydrophobic interactions residues	π stacking
		Residues	Distance (Å)		Donar angle [°]		
			H–A	D–A			
VXY	− 8.7	Arg 770	2.97	3.90	157.36	Ile 848	
		Ser 774	2.34	3.20	148.88		
		Ser 774	2.22	3.20	161.99		
		Lys 802	3.01	4.01	166.90		
		Lys 802	3.45	4.01	117.70		
		Val 851	1.78	2.77	174.60		
		Gln 859	2.08	3.06	171.82		
		Ser 919	1.86	2.77	157.59		
		Asp 933	2.71	3.31	119.48		
Alpelisib	− 11.2	Val 851	2.34	3.24	149.83	Trp 780	
		Val 851	2.08	3.01	156.70	Ile 800	
		Ser 854	2.81	3.63	142.60	Tyr 836	
		Thr 856	3.40	4.10	129.29	Ile 848	
		Gln 859	2.03	2.98	159.70	Val 851	
		Gln 859	2.14	2.94	135.11	Phe 930	
						Ile 932	
Copanlisib	− 9.5	Lys 802	2.92	3.92	167.75	Tyr 836	
		Val 851	1.98	2.96	173.09	Ile 848	
		Asn 853	2.54	3.10	116.39	Ile 932	
		Ser 854	2.63	3.31	126.23	Trp 780	
		Asp 933	2.85	3.44	119.07	Ile 848	
Chloramphenicol	− 7.1	Ser 774	2.04	3.01	174.21	Ile 848	
		Ser 774	2.03	3.01	159.63	Thr 856	
						Ile 932	

We performed similar docking experiments on the crystallographic structure of PI3K gamma (PDB code 8SC8) to assess the binding mode of the studied ligand to this target. In this case, we compared the interaction of chloramphenicol to that of the crystallographic ligand D0D—(*R*)-N-(2-chloro-5-(4-((1-phenylethyl)amino)quinazolin-6-yl)pyridin-3-yl)methanesulfonamide—and the known inhibitors idelalisib and duvelisib.

**FIGURE 10:**
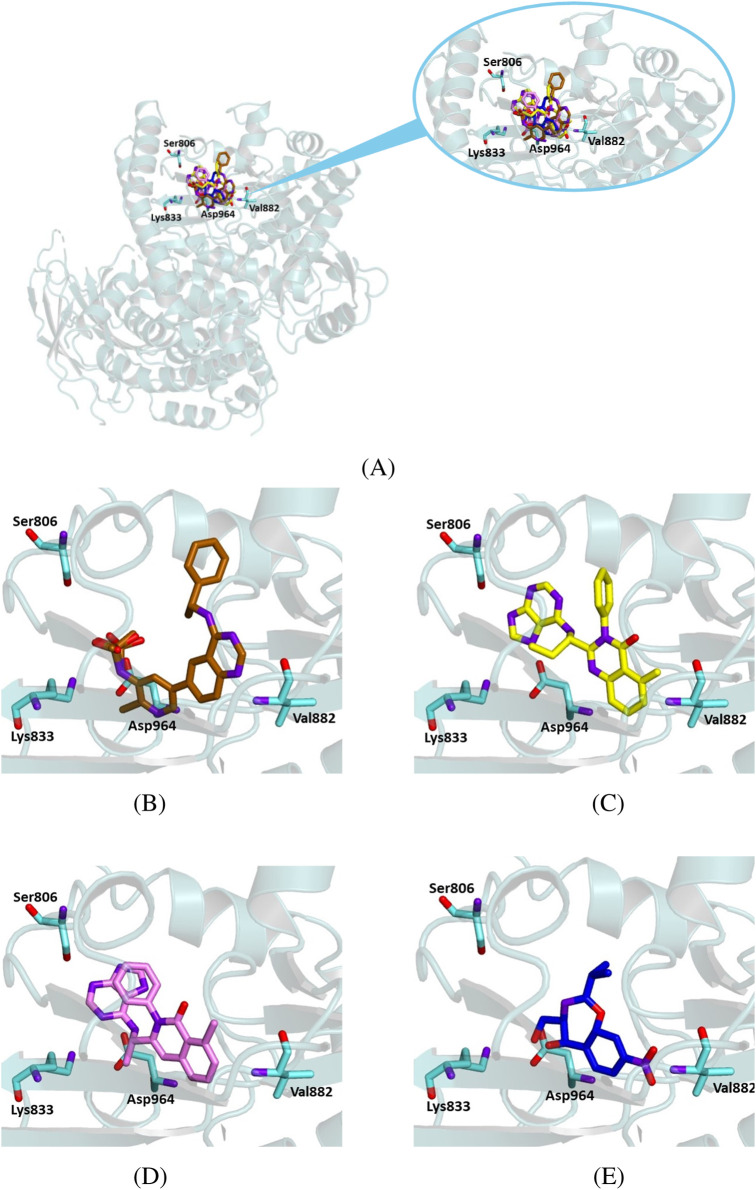
Crystallographic structure of PI3K gamma (PDB 78SC8). The protein backbone is represented as ribbons and key amino acid residues of the catalytic site are in cyan. **(A)** Superimposed binding modes of the crystallographic ligand D0D (gold), idelalisib (yellow), duvelisib (violet), and chloramphenicol (blue). **(B)** The specific binding mode of D0D. **(C)** The specific binding mode of idelalisib. **(D)** The specific binding mode of duvelisib. **(E)** The specific binding mode of chloramphenicol.

Our simulation experiments demonstrated that all the ligands occupied the same region as the crystallographic ligand, corresponding to the enzyme’s catalytic subunit (Figure 10). Chloramphenicol interacted with key residues of the protein active site, although its binding energy values were less favorable than those observed for idelalisib and duvelisib (Table 5).

**TABLE 5:** Binding energy values for ligands complexed with the PI3K gamma catalytic subunit and key protein residues interacting with the ligands.

Ligand	Binding energy [Kcal/mol]	Interactions					
		Hydrogen bonds				Hydrophobic interactions residues	π stacking
		Residues	Distance (Å)		Donar angle [°]		
			H–A	D–A			
D0D	− 10.4	Lys 833	2.65	3.48	137.85	Ile 831	
		Lys 833	2.34	3.27	151.10	Ile 879	
		Val 882	1.96	2.83	144.98	Thr 887	
		Asp 964	2.46	3.28	139.73	Ile 963	
Idelalisib	− 8.9	Ser 806	2.45	2.97	113.29	Pro 810	Tyr 867
		Lys 833	3.52	4.07	115.41	Trp 812	
		Lys 833	3.65	4.07	107.34	Ile 831	
						Ile 879	
						Val 882	
						Phe 961	
						Ile 963	
Duvelisib	− 9.0	Ser 806	2.35	2.80	107.84	Pro 810	Tyr 867
		Lys 833	2.64	3.26	118.99	Ile 831	
		Asp 964	2.24	3.01	131.57	Ile 879	
		Asp 964	2.99	3.83	149.41	Ile 963	
						Asp 964	
Chloramphenicol	− 6.7	Lys 833	2.62	3.22	117.37	Ile 831	Tyr 867
		Lys 833	2.77	3.22	108.73	Ile 879	
						Ile 963	

To further assess the reliability of our experiments, we also tested the interaction between chloramphenicol and the PI3K delta isoform. We docked chloramphenicol with the kinase catalytic subunit of the protein (PDB code 5M6U). Its binding mode was compared to that of the crystallographic ligand 7 KA—(*S*)-2-(1-((6-amino-5-ethynylpyrimidin-4-yl)amino)ethyl)-3-phenylpyrrolo[2,1-*f*][1,2,4]triazin-4(3*H*)-one. For a better perspective, we also docked the known inhibitors idelalisib, duvelisib and copanlisib with the selected protein structure.

**FIGURE 11:**
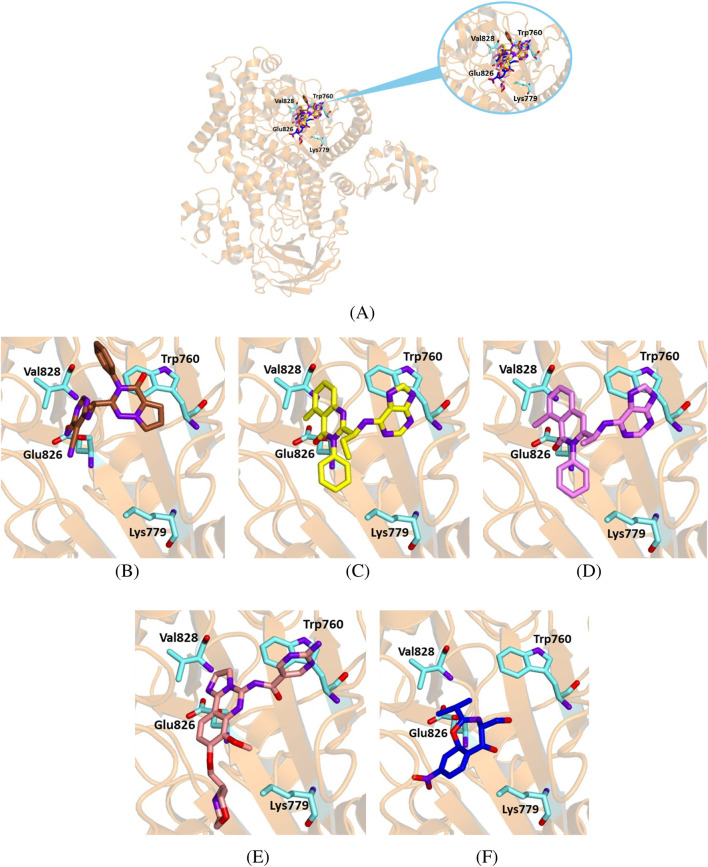
Crystallographic structure of PI3K delta corresponding to the PDB entry 5M6U. The protein backbone is represented in the background as ribbons and the key amino acid residues of the catalytic site are in cyan. **(A)** Superimposed binding modes of the crystallographic ligand 7KA (brown), idelalisib (yellow), duvelisib (violet), copanlisib (salmon) and chloramphenicol (blue). **(B)** The specific binding modes of 7KA. **(C)** The specific binding mode of idelalisib. **(D)** The specific binding mode of duvelisib. **(E)** The specific binding mode of copanlisib. **(F)** The specific binding mode of chloramphenicol.

Chloramphenicol shared a similar orientation within the active site as the known ligands and interacted with key residues for the catalytic activity (Figure 11). Furthermore, the binding energy value for the chloramphenicol-5M6U complex was comparable to that calculated for the crystallographic ligand, although less favorable than those observed for the known inhibitors (Table 6).

**TABLE 6:** Binding energy values for ligands complexed with the PI3K delta catalytic subunit and key protein residues interacting with the ligands.

Ligand	Binding energy [Kcal/mol]	Interactions					
		Hydrogen bonds				Hydrophobic interactions residues	π stacking
		Residues	Distance (Å)		Donar angle [°]		
			H–A	D–A			
7 KA	− 7.6	Tyr 813	3.37	3.67	100.89	Met 752	
		Glu 826	2.13	2.77	121.37	Pro 758	
		Val 828	2.11	3.08	169.52	Trp 760	
						Ile 910	
Idelalisib	− 8.8	Phe 751	3.26	3.78	113.70	Trp 760	Trp 760
		Met 752	3.48	3.88	107.04	Ile 777	
						Lys 779	
						Ile 825	
						Val 828	
						Asp 911	
Duvelisib	− 9.1	Phe 751	3.23	3.71	111.25	Trp 760	Trp 760
		Met 752	3.46	3.86	107.19	Ile 777	
						Lys 779	
						Tyr 813	
						Ile 825	
						Val 828	
						Phe 908	
						Asp 911	
Copanlisib	− 7.4	Asp 911	2.82	3.57	129.08	Met 752	
						Trp 760	
						Lys 779	
						Leu 784	
						Tyr 813	
						Ile 825	
						Ile 910	
Chloramphenicol	− 6.9					Trp 760	
						Ile 777	
						Lys 779	
						Ile 825	
						Ile 910	
						Asp 911	

## 6 Discussions and conclusion

This study demonstrates the potential impact of our network-based pipeline in drug repositioning efforts. Our Drug-Drug Similarity Network (DDSN) generates 34 clusters, which we filtered based on connectivity and cluster size. As such, we focus on the remaining 12 robust clusters for a more detailed analysis (see Figure 3). Our procedure yields a 53.4% success rate in directly matching drugs to their cluster’s level 1 ATC code through DrugBank (Table 2). The literature confirmed pharmacological properties corresponding to ATC level 1 for an additional 20.2% of drugs, thus increasing the prediction accuracy to 73.6% (see GitHub results). We consider the 26.4% of drugs that—according to our current knowledge—do not comply with their assigned cluster label as repositioning candidates. These findings indicate that our network-based pipeline can identify drugs with potential new uses, guiding experimental validation efforts.

Our ATC-based analysis further refines this approach by mapping ATC level 1, 2, 3, and 4 codes. As presented in Figure 4, for Cluster 1, labeled N–*Nervous System*, the hierarchical breakdown reveals dominant drug categories at levels 2, 3, and 4. Extracting nervous system-related targets from DrugBank for level 4 ATC codes helps molecular docking as a validation step, based on the detailed mechanistic insights provided by level 4 ATC codes. Also, in Cluster 6, 60.8% of drugs are L–*Antineoplastic and immunomodulating agents*. Our multi-level ATC approach presents the dominant distribution across level 2 (L01, L04, L03), level 3 (L01E, L04A) and level 4 (L04AA, L01FX, L01XX, L01EM, L01EL) codes (Figure 5). In this way, our pipeline enables the identification of various targets relevant to cancer therapies, including PI3K and BTK1, and proposes their testing to reposition candidates from Cluster 6. As a result, repositioning candidates can be tested with molecular docking, which simulates drug-target interactions and assesses the free energy of binding ( Δ G) Issa et al. (2021); Udrescu et al. (2014); thus, the drugs are prioritized for the active binding site of the target Issa et al. (2021). The most favorable docking-based candidates can be further tested *in vitro* and *in vivo*.

### 6.1 Recovered repositionings

Our automated literature analysis revealed that 20.2% drugs exhibit pharmacological properties aligned with their assigned ATC level 1 category, although their ATC labels according to DrugBank do not match their community labels. For all such cases, the literature provides experimental or clinical evidence supporting the drugs’ mechanisms of action, therapeutic applications, or pharmacological effects that correspond to their respective ATC code labeling.

Here, we provide several examples of drugs for which our repositioning method recovers pharmacological properties confirmed by the literature beyond those assigned by ATC codes. Sildenafil is a versatile molecule with famous repositioning stories, from vasodilator and platelet aggregation inhibitor to penile erection and later vasodilator in pulmonary arterial hypertension Jourdan et al. (2020). DrugBank lists sildenafil in the G–*Genito urinary system and sex hormones* category as a urological drug used to treat erectile dysfunction. In our drug-drug similarity network, sildenafil belongs to Cluster 1, labeled as N–*Nervous system*. Indeed, Xiong and Wintermark review the clinical evidence for the effects of sildenafil on the extent of brain injury, myelination neuroinflammation, and brain function in adults and neonates; they also indicate the clinical trials that test the effects of sildenafil seen in animal models in human newborns and after birth asphyxia Xiong and Wintermark (2022). In addition, another review presents *in vitro* and mouse studies, systematic review, and pilot patient studies reporting the effectiveness of sildenafil in Alzheimer’s disease Sanders (2020).

Spironolactone is another example of how the literature confirms the repositionings recovered by our methodology. Spironolactone is an anti-aldosterone diuretic, officially included in the category of C–*Cardiovascular system* drugs (i.e., C is its first ATC level). Our repositioning method places spironolactone within Cluster 5, which has the ATC level 1 label D–*Dermatologicals*. The review articles by Aguilar Medina et al. (2022); Searle et al. (2020) confirm the beneficial effects of spironolactone in androgen-mediated skin conditions, such as hidradenitis suppurativa, acne, alopecia pattern in women and hirsutism; they also highlight that spironolactone is well tolerated and has a favorable safety profile, i.e., it has few adverse effects, at doses ranging from 25 to 200 mg/day.

One more example of recovered repositionings, amitriptyline, traditionally classified as an antidepressant, is found by our method in cluster L—*Antineoplastic and immunomodulating agents*. In fact, amitriptyline has potential in cancer treatment through various mechanisms. For example, in multiple myeloma (MM) xenograft models, amitriptyline decreases tumor growth and prolongs survival by inducing p53, activating caspase-3, and reducing the anti-apoptotic proteins Bcl-2 and Mcl-1 Zhang et al. (2013). In colorectal cancer cells, amitriptyline and other tricyclic antidepressants reduce cell viability in a time-dependent manner Arimochi and Morita (2006). Furthermore, it inhibits cyclin D2 transactivation, arrests the cell cycle in G0/G1, and modulates histone acetylation by downregulating HDACs, particularly HDAC7, thus enhancing tumor suppressor gene expression Mao et al. (2011). Amitriptyline promotes TRAIL-mediated apoptosis by enhancing the expression of death receptors and caspase activation; it also suppresses autophagy, disrupts lysosomal-autophagosome fusion, and reduces oxidative stress markers, underscoring its antitumor properties Zheng et al. (2023).

Recovered repositioning examples, Table 2 (literature validation, showing the number of drugs per community with literature-confirmed ATC changes), and the exhaustive per-community target lists in the Repositioning hints and predicted targets table in our GitHub results allow readers to explore alternative candidates from other communities.

### 6.2 Molecular docking

A promising result of our approach is the identification of chloramphenicol in Cluster 6. Chloramphenicol is a widely used antibacterial agent, but because it is in cluster 6, it represents a candidate for cancer repositioning. We perform molecular docking simulations to further analyze our computational predictions. Several previous studies have reported anticancer effects of chloramphenicol or its derivatives, including cytotoxicity in mesothelioma cells Giannopoulou et al. (2019), growth inhibition of T-leukemic cells by polyamine-conjugated derivatives Kostopoulou et al. (2015), and apoptosis induction in multiple myeloma cells through mitochondrial protein synthesis inhibition Tian et al. (2016). Furthermore, mitochondria-targeting antibiotics such as chloramphenicol have been shown to eradicate cancer stem cells across tumor types Lamb et al. (2015). However, no experimental binding studies have so far demonstrated a direct interaction of chloramphenicol with BTK1 or PI3K (alpha, gamma, and delta isoforms). Our docking simulations therefore provide the first *in silico* evidence that the drug may interact with these targets, suggesting a dual mechanistic hypothesis combining mitochondrial effects with kinases modulation, which is consistent with its reported anticancer activity and warrants future biochemical validation.

Our docking results indicate that chloramphenicol binds within the active site of BTK1 similarly to ibrutinib, a BTK1 inhibitor. Although chloramphenicol has a lower binding energy than ibrutinib, its complex is stabilized by key hydrogen bonds and hydrophobic interactions (Figure 8; Table 3), suggesting potential kinase inhibitory activity. Similarly, molecular docking of chloramphenicol with PI3K isoforms revealed that the drug binds to the target protein in a manner comparable to that of previously identified inhibitors, such as alpelisib, copanlisib, idelalisib, and duvelisib (Figures 9–11). Although its binding energies are less favorable than those calculated for known inhibitors, chloramphenicol interacts with key amino acid residues through hydrogen bonds and van der Waals forces (Tables 4, 5, 6), supporting its potential role in PI3K modulation. Future studies are required to determine whether chloramphenicol can be repurposed for cancer treatment, offering a cost-effective and adaptable therapeutic option.

### 6.3 Limitations—Edge cases and contradictory evidence

Because the drug-drug similarity network projection treats a drug-gene edge as evidence of a pharmacological relationship without encoding the precise action (agonist, antagonist, substrate, *etc.*), a predicted repositioning can reflect either a potentially beneficial effect or an adverse/off-target effect on the same physiological system.

As an illustrative edge case, clarithromycin clusters in the Cluster 3–*Cardiovascular system*. Clarithromycin is clinically associated with QT-interval prolongation and increased arrhythmia risk—a well-known cardiovascular liability that counsels caution. Our network analysis nevertheless assigned clarithromycin in Cluster 3 because it is pharmacologically linked (*via* targets and shared-disease connectivity) to targets involved in cardiovascular regulation. One specific hypothesis emerging from the community mapping is a putative interaction with NR3C2 (the mineralocorticoid receptor). Antagonism of NR3C2 underlies the cardioprotective effects of established mineralocorticoid receptor antagonists (MRAs), which reduce heart-failure hospitalisation and cardiovascular death; MRAs are, however, associated with an increased risk of hyperkalaemia Jhund et al. (2024). While there is no conclusive evidence that clarithromycin antagonises NR3C2 *in vivo*, a retrospective clinical observation that clarithromycin co-administered with MRAs is associated with higher serum potassium levels provides indirect, functional context for an interaction with the mineralocorticoid system Hirai et al. (2023). Taken together, these lines of evidence make NR3C2 a biologically plausible target for further mechanistic investigation. However, any therapeutic hypothesis should be experimentally validated and carefully evaluated against clarithromycin’s known cardiac risks.

### 6.4 Future work

This study highlights the potential of our DDSN-based drug repositioning pipeline to identify new targets for existing drugs and facilitate molecular docking investigations. The pipeline accurately aligns 73.6% of the drugs with their cluster’s dominant level 1 ATC property and classifies the non-conforming remainder as repositioning candidates; overall, this means a good drug property prediction accuracy given the extent of unknown information. Furthermore, our automated pipeline also identifies biological targets that correspond to the majority of drugs within a cluster and proposes them as potential new targets for the repositioning of candidates.

In future studies, we can extend our definition of drug-drug similarity to simplicial complexes (which generalize the notion of graph by allowing higher-dimensional relationships between nodes, not just pairwise edges).

Future work may also validate the repositioning candidates identified by our method (see our GitHub results), by applying a combination of dry-lab approaches (i.e., molecular docking simulations) and wet-lab experiments (i.e., *in vitro* and *in vivo* tests) to confirm their new pharmacological properties and therapeutic potential.

## Data Availability

The original contributions presented in the study are included in the article and via the following link: https://github.com/GrozaVlad/Drug-repurposing-using-DDSN-with-disgenet. Further inquiries can be directed to the corresponding authors.

## References

[B1] Aguilar Medina D. A. Cazarín J. Magaña M. (2022). Spironolactone in dermatology. Dermatol. Ther. 35, e15321. 10.1111/dth.15321 35038224

[B2] Ahmed F. Lee J. W. Samantasinghar A. Kim Y. S. Kim K. H. Kang I. S. (2022). Speropredictor: an integrated machine learning and molecular docking-based drug repurposing framework with use case of COVID-19. Front. public health 10, 902123. 10.3389/fpubh.2022.902123 35784208 PMC9244710

[B3] Al Hasan M. Sabirianov M. Redwine G. Goettsch K. Yang S. X. Zhong H. A. (2023). Binding and selectivity studies of phosphatidylinositol 3-kinase (pi3k) inhibitors. J. Mol. Graph. Model. 121, 108433. 10.1016/j.jmgm.2023.108433 36812742

[B4] Alam M. S. Rahaman M. M. Sultana A. Wang G. Mollah M. N. H. (2022). Statistics and network-based approaches to identify molecular mechanisms that drive the progression of breast cancer. Comput. Biol. Med. 145, 105508. 10.1016/j.compbiomed.2022.105508 35447458

[B5] Amadori L. Calcagno C. Fernandez D. M. Koplev S. Fernandez N. Kaur R. (2023). Systems immunology-based drug repurposing framework to target inflammation in atherosclerosis. Nat. Cardiovasc. Res. 2, 550–571. 10.1038/s44161-023-00278-y 37771373 PMC10538622

[B6] Amiri Souri E. Laddach R. Karagiannis S. Papageorgiou L. G. Tsoka S. (2022). Novel drug-target interactions *via* link prediction and network embedding. BMC Bioinforma. 23, 121. 10.1186/s12859-022-04650-w 35379165 PMC8978405

[B7] Arimochi H. Morita K. (2006). Characterization of cytotoxic actions of tricyclic antidepressants on human ht29 colon carcinoma cells. Eur. J. Pharmacol. 541, 17–23. 10.1016/j.ejphar.2006.04.053 16753142

[B8] Bender A. T. Gardberg A. Pereira A. Johnson T. Wu Y. Grenningloh R. (2017). Ability of bruton’s tyrosine kinase inhibitors to sequester y551 and prevent phosphorylation determines potency for inhibition of fc receptor but not b-cell receptor signaling. Mol. Pharmacol. 91, 208–219. 10.1124/mol.116.107037 28062735

[B9] Bender B. J. Gahbauer S. Luttens A. Lyu J. Webb C. M. Stein R. M. (2021). A practical guide to large-scale docking. Nat. Protoc. 16, 4799–4832. 10.1038/s41596-021-00597-z 34561691 PMC8522653

[B10] Brown A. S. Kong S. W. Kohane I. S. Patel C. J. (2016). Ksrepo: a generalized platform for computational drug repositioning. BMC Bioinforma. 17, 78–5. 10.1186/s12859-016-0931-y 26860211 PMC4746802

[B11] Cheng H. Orr S. T. Bailey S. Brooun A. Chen P. Deal J. G. (2020). Structure-based drug design and synthesis of pi3k*α*-selective inhibitor (pf-06843195). J. Med. Chem. 64, 644–661. 10.1021/acs.jmedchem.0c01652 33356246

[B12] Cock P. J. Antao T. Chang J. T. Chapman B. A. Cox C. J. Dalke A. (2009). Biopython: freely available python tools for computational molecular biology and bioinformatics. Bioinformatics 25, 1422–1423. 10.1093/bioinformatics/btp163 19304878 PMC2682512

[B13] Conte F. Sibilio P. Fiscon G. Paci P. (2022). A transcriptome-and interactome-based analysis identifies repurposable drugs for human breast cancer subtypes. Symmetry 14, 2230. 10.3390/sym14112230

[B14] Erra M. Taltavull J. Gréco A. Bernal F. J. Caturla J. F. Gràcia J. (2017). Discovery of a potent, selective, and orally available pi3k*δ* inhibitor for the treatment of inflammatory diseases. ACS Med. Chem. Lett. 8, 118–123. 10.1021/acsmedchemlett.6b00438 28105286 PMC5238472

[B15] Fadlalla M. Ahmed M. Ali M. Elshiekh A. A. Yousef B. A. (2022). Molecular docking as a potential approach in repurposing drugs against COVID-19: a systematic review and novel pharmacophore models. Curr. Pharmacol. Rep. 8, 212–226. 10.1007/s40495-022-00285-w 35381996 PMC8970976

[B16] Fetro C. Scherman D. (2020). Drug repurposing in rare diseases: myths and reality. Therapies 75, 157–160. 10.1016/j.therap.2020.02.006 32241561

[B17] Fiscon G. Paci P. (2021). Saverunner: an r-based tool for drug repurposing. BMC Bioinforma. 22, 150–10. 10.1186/s12859-021-04076-w 33757425 PMC7987121

[B18] Fiscon G. Conte F. Amadio S. Volonté C. Paci P. (2021a). Drug repurposing: a network-based approach to amyotrophic lateral sclerosis. Neurotherapeutics 18, 1678–1691. 10.1007/s13311-021-01064-z 33987813 PMC8609089

[B19] Fiscon G. Conte F. Farina L. Paci P. (2021b). Saverunner: a network-based algorithm for drug repurposing and its application to COVID-19. PLoS Comput. Biol. 17, e1008686. 10.1371/journal.pcbi.1008686 33544720 PMC7891752

[B20] Giannopoulou P. C. Missiri D. A. Kournoutou G. G. Sazakli E. Papadopoulos G. E. Papaioannou D. (2019). New chloramphenicol derivatives from the viewpoint of anticancer and antimicrobial activity. Antibiotics 8, 9. 10.3390/antibiotics8010009 30699905 PMC6466596

[B21] Grande F. Occhiuzzi M. A. Lappano R. Cirillo F. Guzzi R. Garofalo A. (2020). Computational approaches for the discovery of gper targeting compounds. Front. Endocrinol. 11, 517. 10.3389/fendo.2020.00517 32849301 PMC7417359

[B22] Grande F. Occhiuzzi M. A. Perri M. R. Ioele G. Rizzuti B. Statti G. (2021). Polyphenols from citrus tacle® extract endowed with hmgcr inhibitory activity: an antihypercholesterolemia natural remedy. Molecules 26, 5718. 10.3390/molecules26185718 34577189 PMC8470345

[B23] Groza V. Udrescu M. Bozdog A. Udrescu L. (2021). Drug repurposing using modularity clustering in drug-drug similarity networks based on drug–gene interactions. Pharmaceutics 13, 2117. 10.3390/pharmaceutics13122117 34959398 PMC8709282

[B24] Hanwell M. D. Curtis D. E. Lonie D. C. Vandermeersch T. Zurek E. Hutchison G. R. (2012). Avogadro: an advanced semantic chemical editor, visualization, and analysis platform. J. Cheminformatics 4, 17. 10.1186/1758-2946-4-17 22889332 PMC3542060

[B25] Hirai T. Ueda S. Ogura T. Katayama K. Dohi K. Hosohata K. (2023). Hyperkalemia by eplerenone or esaxerenone in the presence or absence of clarithromycin in hypertensive patients: a retrospective observational cohort study. J. Hypertens. 41, 580–586. 10.1097/hjh.0000000000003372 36655800

[B26] Issa N. T. Stathias V. Schürer S. Dakshanamurthy S. (2021). Machine and deep learning approaches for cancer drug repurposing. Seminars Cancer Biol. 68, 132–142. 10.1016/j.semcancer.2019.12.011 31904426 PMC7723306

[B27] Jarada T. N. Rokne J. G. Alhajj R. (2020). A review of computational drug repositioning: strategies, approaches, opportunities, challenges, and directions. J. Cheminformatics 12, 46–23. 10.1186/s13321-020-00450-7 33431024 PMC7374666

[B28] Jhund P. S. Talebi A. Henderson A. D. Claggett B. L. Vaduganathan M. Desai A. S. (2024). Mineralocorticoid receptor antagonists in heart failure: an individual patient level meta-analysis. Lancet 404, 1119–1131. 10.1016/s0140-6736(24)01733-1 39232490

[B29] Jourdan J.-P. Bureau R. Rochais C. Dallemagne P. (2020). Drug repositioning: a brief overview. J. Pharm. Pharmacol. 72, 1145–1151. 10.1111/jphp.13273 32301512 PMC7262062

[B30] Ko Y. (2020). Computational drug repositioning: current progress and challenges. Appl. Sci. 10, 5076. 10.3390/app10155076

[B31] Kostopoulou O. N. Magoulas G. E. Papadopoulos G. E. Mouzaki A. Dinos G. P. Papaioannou D. (2015). Synthesis and evaluation of chloramphenicol homodimers: molecular target, antimicrobial activity, and toxicity against human cells. PLoS One 10, e0134526. 10.1371/journal.pone.0134526 26267355 PMC4533973

[B32] Lamb R. Ozsvari B. Lisanti C. L. Tanowitz H. B. Howell A. Martinez-Outschoorn U. E. (2015). Antibiotics that target mitochondria effectively eradicate cancer stem cells, across multiple tumor types: treating cancer like an infectious disease. Oncotarget 6, 4569–4584. 10.18632/oncotarget.3174 25625193 PMC4467100

[B33] Liu R. Wei L. Zhang P. (2021). A deep learning framework for drug repurposing *via* emulating clinical trials on real-world patient data. Nat. Mach. Intell. 3, 68–75. 10.1038/s42256-020-00276-w 35603127 PMC9119409

[B34] Luo Y. Zhao X. Zhou J. Yang J. Zhang Y. Kuang W. (2017). A network integration approach for drug-target interaction prediction and computational drug repositioning from heterogeneous information. Nat. Commun. 8, 573. 10.1038/s41467-017-00680-8 28924171 PMC5603535

[B35] Lv X. Wang J. Yuan Y. Pan L. Liu Q. Guo J. (2024). *In silico* drug repurposing pipeline using deep learning and structure based approaches in epilepsy. Sci. Rep. 14, 16562. 10.1038/s41598-024-67594-6 39020064 PMC11254927

[B36] Mangione W. Falls Z. Chopra G. Samudrala R. (2020). Cando. py: open source software for predictive bioanalytics of large scale drug–protein–disease data. J. Chem. Inf. Model. 60, 4131–4136. 10.1021/acs.jcim.0c00110 32515949 PMC8098009

[B37] Mangione W. Falls Z. Samudrala R. (2022). Optimal COVID-19 therapeutic candidate discovery using the cando platform. Front. Pharmacol. 13, 970494. 10.3389/fphar.2022.970494 36091793 PMC9452636

[B38] Mao X. Hou T. Cao B. Wang W. Li Z. Chen S. (2011). The tricyclic antidepressant amitriptyline inhibits d-cyclin transactivation and induces myeloma cell apoptosis by inhibiting histone deacetylases: *in vitro* and *in silico* evidence. Mol. Pharmacol. 79, 672–680. 10.1124/mol.110.068122 21220410

[B39] Minadakis G. Tomazou M. Dietis N. Spyrou G. M. (2023). Vir2drug: a drug repurposing framework based on protein similarities between pathogens. Briefings Bioinforma. 24, bbac536. 10.1093/bib/bbac536 36513376 PMC9851336

[B40] Morris G. M. Goodsell D. S. Halliday R. S. Huey R. Hart W. E. Belew R. K. (1998). Automated docking using a lamarckian genetic algorithm and an empirical binding free energy function. J. Comput. Chem. 19, 1639–1662. 10.1002/(SICI)1096-987X(19981115)19:14⟨1639::AID-JCC10⟩3.0.CO;2-B

[B41] Morselli Gysi D. Do Valle Í. Zitnik M. Ameli A. Gan X. Varol O. (2021). Network medicine framework for identifying drug-repurposing opportunities for COVID-19, Proc. Natl. Acad. Sci. U. S. A. 118, e2025581118. 10.1073/pnas.2025581118 33906951 PMC8126852

[B42] Paci P. Fiscon G. Conte F. Wang R.-S. Handy D. E. Farina L. (2022). Comprehensive network medicine-based drug repositioning *via* integration of therapeutic efficacy and side effects. NPJ Syst. Biol. Appl. 8, 12. 10.1038/s41540-022-00221-0 35443763 PMC9021283

[B43] Pacini C. Iorio F. Gonçalves E. Iskar M. Klabunde T. Bork P. (2013). Dvd: an r/cytoscape pipeline for drug repurposing using public repositories of gene expression data. Bioinformatics 29, 132–134. 10.1093/bioinformatics/bts656 23129297 PMC3530913

[B44] Pal Singh S. Dammeijer F. Hendriks R. W. (2018). Role of bruton’s tyrosine kinase in b cells and malignancies. Mol. Cancer 17, 57. 10.1186/s12943-018-0779-z 29455639 PMC5817726

[B45] Parvathaneni V. Kulkarni N. S. Muth A. Gupta V. (2019). Drug repurposing: a promising tool to accelerate the drug discovery process. Drug Discov. Today 24, 2076–2085. 10.1016/j.drudis.2019.06.014 31238113 PMC11920972

[B46] Perri M. R. Pellegrino M. Marrelli M. Aquaro S. Cavaliere F. Grande F. (2023). Identification of pinosylvin in pinus nigra subsp. laricio: a naturally occurring stilbenoid suppressing lps-induced expression of pro-inflammatory cytokines and mediators and inhibiting the jak/stat signaling pathway. Pharmaceuticals 16, 718. 10.3390/ph16050718 37242501 PMC10221723

[B47] Pham D.-T. Tran T.-D. (2024). Drivergene. net: a cytoscape app for the identification of driver nodes of large-scale complex networks and case studies in discovery of drug target genes. Comput. Biol. Med. 179, 108888. 10.1016/j.compbiomed.2024.108888 39047507

[B48] Piñero J. Ramírez-Anguita J. M. Saüch-Pitarch J. Ronzano F. Centeno E. Sanz F. (2020). The disgenet knowledge platform for disease genomics: 2019 update. Nucleic acids Res. 48, D845–D855. 10.1093/nar/gkz1021 31680165 PMC7145631

[B49] Pushpakom S. Iorio F. Eyers P. A. Escott K. J. Hopper S. Wells A. (2019). Drug repurposing: progress, challenges and recommendations. Nat. Rev. Drug Discov. 18, 41–58. 10.1038/nrd.2018.168 30310233

[B50] Salentin S. Schreiber S. Haupt V. J. Adasme M. F. Schroeder M. (2015). Plip: fully automated protein–ligand interaction profiler. Nucleic Acids Res. 43, W443–W447. 10.1093/nar/gkv315 25873628 PMC4489249

[B51] Sanders O. (2020). Sildenafil for the treatment of alzheimer’s disease: a systematic review. J. Alzheimer’s Dis. Rep. 4, 91–106. 10.3233/ADR-200166 32467879 PMC7242821

[B52] Searle T. Al-Niaimi F. Ali F. R. (2020). Spironolactone in dermatology: uses in acne and beyond. Clin. Exp. Dermatol. 45, 986–993. 10.1111/ced.14340 32844462

[B53] Sharma P. P. Bansal M. Sethi A. Pena L. Goel V. K. Grishina M. (2021). Computational methods directed towards drug repurposing for COVID-19: advantages and limitations. RSC Adv. 11, 36181–36198. 10.1039/D1RA05320E 35492747 PMC9043418

[B54] Shuey M. M. Lee K. M. Keaton J. Khankari N. K. Breeyear J. H. Walker V. M. (2023). A genetically supported drug repurposing pipeline for diabetes treatment using electronic health records. EBioMedicine 94, 104674. 10.1016/j.ebiom.2023.104674 37399599 PMC10328805

[B55] Smith A. G. Dovey G. Cartwright R. (2000). Topical chloramphenicol and the risk of acute leukaemia in adults. Pharmacoepidemiol. Drug Saf. 9, 215–219. 10.1002/1099-1557(200005/06)9:3<215::aid-pds497>3.0.co;2-k 19025822

[B56] Tian F. Wang C. Tang M. Li J. Cheng X. Zhang S. (2016). The antibiotic chloramphenicol may be an effective new agent for inhibiting the growth of multiple myeloma. Oncotarget 7, 51934–51942. 10.18632/oncotarget.10623 27437770 PMC5239525

[B57] Tian Z. Teng Z. Cheng S. Guo M. (2018). Computational drug repositioning using meta-path-based semantic network analysis. BMC Syst. Biol. 12, 134. 10.1186/s12918-018-0658-7 30598084 PMC6311940

[B58] Traylor J. I. Sheppard H. E. Ravikumar V. Breshears J. Raza S. M. Lin C. Y. (2021). Computational drug repositioning identifies potentially active therapies for chordoma. Neurosurgery 88, 428–436. 10.1093/neuros/nyaa398 33017025 PMC7803434

[B59] Trott O. Olson A. J. (2010). Autodock vina: improving the speed and accuracy of docking with a new scoring function, efficient optimization, and multithreading. J. Comput. Chem. 31, 455–461. 10.1002/jcc.21334 19499576 PMC3041641

[B60] Tuerkova A. Zdrazil B. (2020). A ligand-based computational drug repurposing pipeline using knime and programmatic data access: case studies for rare diseases and COVID-19. J. cheminformatics 12, 71. 10.1186/s13321-020-00474-z 33250934 PMC7686838

[B61] Tundis R. Grande F. Occhiuzzi M. A. Sicari V. Loizzo M. R. Cappello A. R. (2023). Lavandula angustifolia mill.(lamiaceae) ethanol extract and its main constituents as promising agents for the treatment of metabolic disorders: chemical profile, *in vitro* biological studies, and molecular docking. J. Enzyme Inhibition Med. Chem. 38, 2269481. 10.1080/14756366.2023.2269481 37850338 PMC10586085

[B62] Udrescu L. Sbarcea L. (2020). The new tale of some old drugs,” in Proceedings “17th Romanian national congress oh pharmacy – 21st century pharmacy – between intelligent specialization and social responsibility - 2018, 234–240. Bologna, Italy: Filodiritto

[B63] Udrescu M. Udrescu L. (2019). A drug repurposing method based on drug-drug interaction networks and using energy model layouts. Comput. Methods Drug Repurposing 1903, 185–201. 10.1007/978-1-4939-8955-3_11 30547443

[B64] Udrescu L. Sbârcea L. Fuliaş A. Ledeți I. Vlase G. Barvinschi P. (2014). Physicochemical analysis and molecular modeling of the fosinopril *β*-cyclodextrin inclusion complex. J. Spectrosc. 2014, 748468. 10.1155/2014/748468

[B65] Udrescu L. Sbârcea L. Topîrceanu A. Iovanovici A. Kurunczi L. Bogdan P. (2016). Clustering drug-drug interaction networks with energy model layouts: community analysis and drug repurposing. Sci. Rep. 6, 32745. 10.1038/srep32745 27599720 PMC5013446

[B66] Udrescu L. Bogdan P. Chiş A. Sîrbu I. O. Topîrceanu A. Văruţ R.-M. (2020). Uncovering new drug properties in target-based drug–drug similarity networks. Pharmaceutics 12, 879. 10.3390/pharmaceutics12090879 32947845 PMC7557376

[B67] Udrescu M. Ardelean S. M. Udrescu L. (2023). The curse and blessing of abundance—the evolution of drug interaction databases and their impact on drug network analysis. GigaScience 12, giad011. 10.1093/gigascience/giad011 36892110 PMC10023830

[B68] Vanhaesebroeck B. Guillermet-Guibert J. Graupera M. Bilanges B. (2010). The emerging mechanisms of isoform-specific pi3k signalling. Nat. Rev. Mol. cell Biol. 11, 329–341. 10.1038/nrm2882 20379207

[B69] Wishart D. S. Feunang Y. D. Guo A. C. Lo E. J. Marcu A. Grant J. R. (2018). Drugbank 5.0: a major update to the drugbank database for 2018. Nucleic Acids Res. 46, D1074–D1082. 10.1093/nar/gkx1037 29126136 PMC5753335

[B70] Wolfram Mathematica 13 Inc. (2023). Mathematica, version 13.3. Champaign, IL.

[B71] Xiong Y. Wintermark P. (2022). The role of sildenafil in treating brain injuries in adults and neonates. Front. Cell. Neurosci. 16, 879649. 10.3389/fncel.2022.879649 35620219 PMC9127063

[B72] Yuan Z.-R. Shi Y. (2008). Chloramphenicol induces abnormal differentiation and inhibits apoptosis in activated t cells. Cancer Res. 68, 4875–4881. 10.1158/0008-5472.CAN-07-6061 18559535

[B73] Zhang Z. Du X. Zhao C. Cao B. Zhao Y. Mao X. (2013). The antidepressant amitriptyline shows potent therapeutic activity against multiple myeloma. Anti-cancer drugs 24, 792–798. 10.1097/CAD.0b013e3283628c21 23708819

[B74] Zhang Z. Zhou L. Xie N. Nice E. C. Zhang T. Cui Y. (2020). Overcoming cancer therapeutic bottleneck by drug repurposing. Signal Transduct. Target. Ther. 5, 113. 10.1038/s41392-020-00213-8 32616710 PMC7331117

[B75] Zheng Y. Chang X. Huang Y. He D. (2023). The application of antidepressant drugs in cancer treatment. Biomed. and Pharmacother. 157, 113985. 10.1016/j.biopha.2022.113985 36402031

